# Preferential encoding of behaviorally relevant predictions revealed by EEG

**DOI:** 10.3389/fnhum.2014.00687

**Published:** 2014-09-02

**Authors:** Mark G. Stokes, Nicholas E. Myers, Jonathan Turnbull, Anna C. Nobre

**Affiliations:** ^1^Department of Experimental Psychology, University of OxfordOxford, UK; ^2^Oxford Centre for Human Brain Activity, University of OxfordOxford, UK

**Keywords:** prediction, expectation, task-relevance, EEG, event-related potential

## Abstract

Statistical regularities in the environment guide perceptual processing; however, some predictions are bound to be more important than others. In this electroencephalogram (EEG) study, we test how task relevance influences the way predictions are learned from the statistics of visual input, and exploited for behavior. We developed a novel task in which participants are simply instructed to respond to a designated target stimulus embedded in a serial stream of non-target stimuli. Presentation probabilities were manipulated such that a designated target cue stimulus predicted the target onset with 70% validity. We also included a corresponding control contingency: a pre-designated control cue predicted a specific non-target stimulus with 70% validity. Participants were not informed about these contingencies. This design allowed us to examine the neural response to task-relevant predictive (cue) and predicted stimuli (target), relative to task-irrelevant predictive (control cue) and predicted stimuli (control non-target). The behavioral results confirmed that participants learned and exploited task-relevant predictions even when not explicitly defined. The EEG results further showed that target-relevant predictions are coded more strongly than statistically equivalent regularities between non-target stimuli. There was a robust modulation of the response for predicted targets associated with learning, enhancing the response to cued stimuli just after 200 ms post-stimulus in central and posterior electrodes, but no corresponding effects for predicted non-target stimuli. These effects of target prediction were preceded by a sustained frontal negativity following presentation of the predictive cue stimulus. These results show that task relevance critically influences how the brain extracts predictive structure from the environment, and exploits these regularities for optimized behavior.

## INTRODUCTION

Past experience provides a powerful constraint for perception. The statistical regularities of the environment bias the focus of attention (Summerfield et al., 2006; Chun and Turk-Browne, 2007) and perceptual inferences (Bar, 2009; Summerfield et al., 2011). However, it is unlikely that all predictions are treated equally: some statistical regularities are simply more important for behavior than others (Brown and Friston, 2013). In this study, we explore neural encoding for predictions that are directly relevant to behavior, compared to identical contingencies that relate to non-target stimuli.

Extensive evidence suggests that the brain actively generates predictions about the environment to optimize behavior (Friston, 2010). In the classic oddball paradigm (Squires et al., 1975), for example, repeated stimuli (standards) generate a reduced neural response relative to an occasional deviant stimulus (oddball stimulus). Although the underlying mechanisms that mediate repetition suppression are still a matter of debate (Krekelberg et al., 2006; De Baene and Vogels, 2010), the essential phenomenon is consistent with the broader theoretical framework of predictive coding (Todorovic and de Lange, 2012). Assuming a relatively stable environment (i.e., temporal autocorrelation), repetition is more expected than not: therefore repetitions contain little new information for guiding behavior. In informational terms, it is more efficient to signal change than constancy. In the predictive coding framework, the enhanced response to change reflects the *prediction errors* that are most informative (Rao and Ballard, 1999). The suppression of expected input effectively reduces redundancy in the neural code.

This framework also extends to more complex learned statistical structure. For example, den Ouden et al. (2009) found that participants learned an arbitrary association between a task-irrelevant auditory stimulus and the presentation of an equally irrelevant visual stimulus (den Ouden et al., 2009). Participants implicitly learned this task-irrelevant predictive relationship, as evidenced by a modulation of visual activity triggered by the predictive auditory stimulus that emerged during the experimental session. Consistent with the predictive coding model, visual activity for the predicted visual stimulus reduced as the predictive relationship was learned. Moreover, violations of the prediction (absent stimuli) elicited an increasingly large response as learning progressed.

Predictive coding emphasizes attenuation of expected input, however, predictions relating to task-relevant information can also be used to enhance perceptual processing. In classic models of selective attention (Desimone and Duncan, 1995), top-down feedback prepares sensory areas for processing expected target stimuli (Moore, 2006). In standard attention tasks, target expectations are often established via explicit symbolic cueing (Posner, 1980), resulting in preparatory activity for the cued feature (e.g., spatial location in Kastner et al., 1999; or non-spatial feature in Stokes et al., 2009). Target expectations can also be established through direct experience, by learning the regularities in the environment (Chun and Turk-Browne, 2007). For example, we (Summerfield et al., 2006; Patai et al., 2012; Stokes et al., 2012) and others (Chun and Jiang, 1998, 2003) have shown that long-term memory can be used to direct attention to expected target locations according to previously learned predictions. In a recent study, predictable stimulus sequences were even shown to draw attention to irrelevant locations within visual search (Zhao et al., 2013). Moreover, memory-guided attention could operate independently of explicit task strategy, as benefits are even observed when predictions are learned implicitly (e.g., Turk-Browne et al., 2010).

Enhancement effects of target expectation clearly contrast with expectation suppression, suggesting that task-relevance mediates how expectation influences processing (Bendixen et al., 2012). Differential consequences of expectation would have obvious behavioral advantages, insofar as different predictions will have different consequences for behavior (Vetter and Newen, 2014). Irrelevant expected input could be suppressed to reduce redundancy in the neural code and reduce potential distractions (den Ouden et al., 2009), whereas predictions of behaviorally relevant events could be used to override expectation suppression to enhance processing in favor of likely targets.

Here we use electroencephalogram (EEG) to test how task relevance interacts with expectations derived from the statistical regularity of a sequence of images. In this task, participants are simply instructed to respond with a button press to a predefined target image, selected randomly from a set of ten fractal images. Unbeknownst to the participant, another image was pre-selected to serve as the “target cue.” This image predicted the subsequent likelihood of a target with 70% validity. Participants learned and exploited this predictive relationship, as evidenced by a reaction time benefit on cued targets relative to uncued targets that developed across the course of the experiment. This behavioral advantage was also reflected in modulations of the EEG response to the predicted target that emerged with learning, and a preparatory modulation in frontal sensors triggered by the learned predictive cue stimulus.

Crucially, we also included a control condition in which two other visual stimuli were preselected at random to serve as a *control* predictive relationship. The presentation probabilities were identical to those used for the target and target-related cue, but were of minimal behavioral relevance to the participant. By definition, this manipulation precludes a behavioral response, therefore we focused on the EEG response to track the consequence of the matched, but behaviorally irrelevant predictive stimuli. We found no evidence for a neural effect of these predictions over time, suggesting that task-relevant predictions are established more robustly than task-irrelevant predictions.

## MATERIALS AND METHODS

### PARTICIPANTS

Twenty-one volunteers (15 females, mean age 21, range 18–28) participated in this study. All were right-handed with normal/corrected-to-normal vision, had no history of neurological disorders, and were not taking any neurological medications. All participants gave informed written consent, and were remunerated £ 20 for their time. The experiment was approved by the Oxford Central University Research Ethics Committee. Three participants with excessive eye-blink artifacts were excluded from all analyses.

### TASK

**FIGURE 1:**
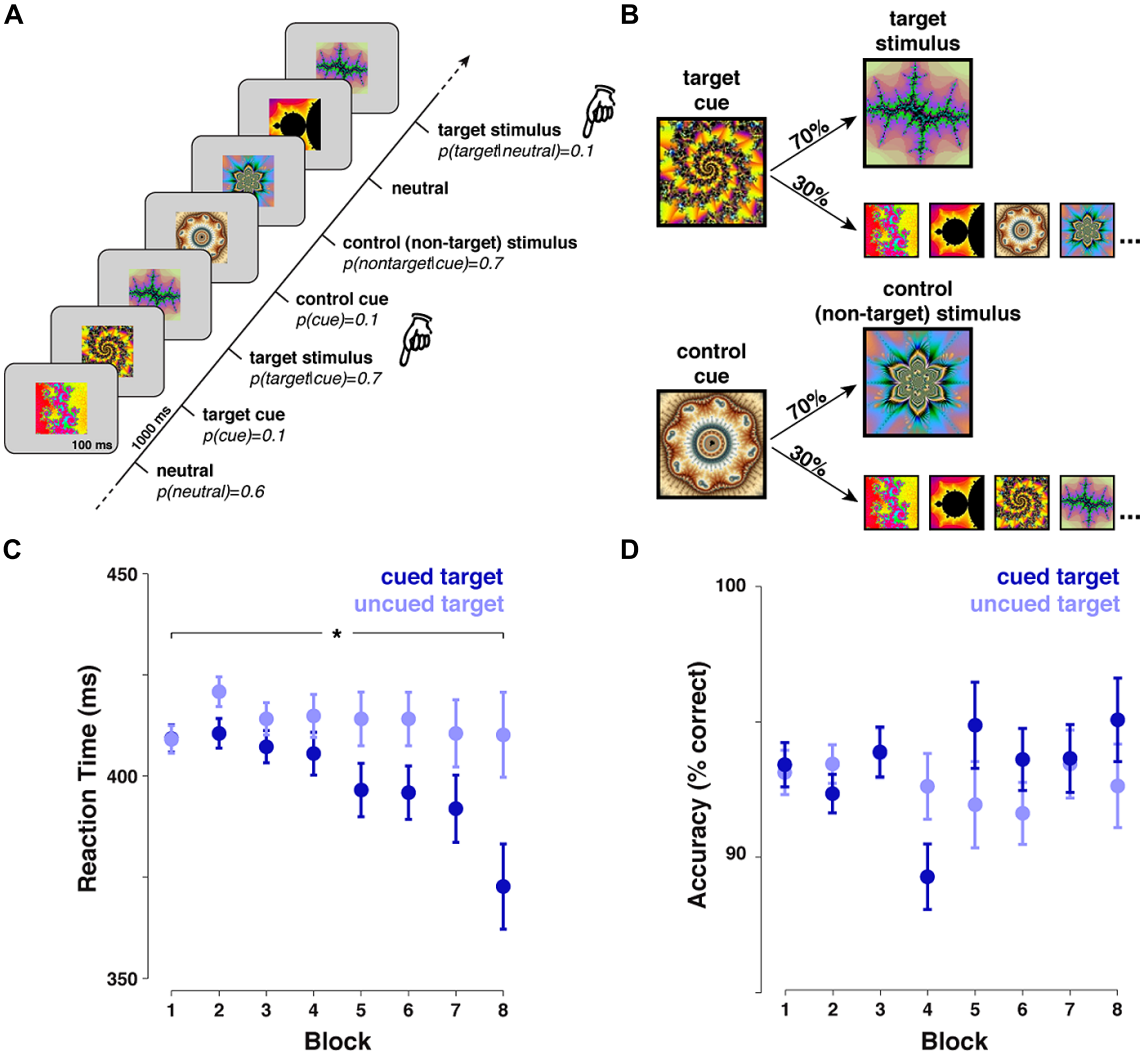
**Task, design and behavioral performance. (A)** Participants viewed a stream of fractal images presented at a rate of one per second at the center of the screen. Their task instruction was simply to press the response button after any presentation of a pre-defined target stimulus as quickly as possible. The target was pre-selected randomly for each participant from a set of 10 fractal images. The participant was only instructed to respond to the target, the nine remaining stimuli could effectively be ignored. **(B)** The presentation probabilities were manipulated. By default, each stimulus was equiprobable, except after a pre-designated target cue (upper) or control cue (lower). These predictive stimuli were followed by the target or control non-target stimulus with 70% validity. On the remaining 30% of trials, stimuli were selected at random from the set of nine remaining stimuli, including the cues and the control or target stimulus. **(C)** The behavioral response to target stimuli indicated that reaction times were faster for cued targets, relative to uncued targets (main effect: *F*_1,17_ = 4.701, *p* = 0.045, interaction with block: *F*_7,119_ = 2.621, *p* = 0.015). **(D)** Accuracy was high overall, and was not modulated by cues (*p* = 0.419).

The task structure is shown in **Figure 1**, along with stimulus probabilities. Participants were shown a sequence of colored fractal images (50 ms duration, 2.08°× 2.08° visual angle) presented against a gray background (RGB: 127,127,127) and separated by a 1000 ms inter-stimulus-interval. For each participant, one fractal image was randomly assigned as the “target” stimulus and one as the “target cue” stimulus. Another fractal was also selected to serve as the “control” non-target stimulus (i.e., presented with the same probability as the real target), and a fourth stimulus for a “control cue” stimulus (i.e., with the same predictive relationship to the control non-target as the relationship between the target cue and target). The remaining six fractal stimuli served as neutral non-target stimuli. Participants were reminded which was the target stimulus at the beginning of each block, but were not informed about any of the other assignments.

The first stimulus in each block was chosen randomly from the neutral condition, however, the next item was drawn from the full set of possibilities (with replacement). The only task for the participant was to press the response key as quickly as possible with their right hand following each presentation of the designated “target” stimulus. The main experimental manipulation resulted in the following presentation probabilities. Following a “*neutral*” non-target the default probability for any stimulus was 10%. Following a “*target cue*” stimulus the probability of “*target”* presentation was raised to 70% (all remaining stimuli were equiprobable). Equivalently, following a “*control cue*” stimulus the probability of the “*control non-target*” stimulus was raised to 70% (all remaining stimuli were equiprobable). With this probability structure, we can directly compare the neural response to predictive target cues against a neutral baseline (cue vs. neutral), and measure the neural consequences of predictions on a target stimulus by comparing cued with uncued targets. These responses, in turn, can be compared to responses to the non-target stimuli with matching probabilities (control cue vs. neutral; cued vs. uncued control non-target).

Participants sat in a dimly-lit booth at a distance of 74 cm from the monitor (22 inch Samsung SyncMaster 2233; resolution: 1680 × 1050 pixels; refresh rate: 60 Hz; screen width: 47 cm). The experimental script was generated dynamically and all stimulus displays delivered via the Psychophysics Toolbox (Brainard, 1997) on MATLAB (version 2011b, The Mathworks Inc., Natick, NA, USA). A chin-rest was used to minimize head movements, and participants were instructed to refrain from excessive blinking and to keep their face as relaxed as possible to avoid muscular artifacts in the EEG recordings. There were 130 trials per run, three runs per block and eight blocks in total. After artifact removal, the average number of trials per sub-condition was as follows: 135/144 cued/uncued targets; 140/156 cued/ uncued control non-target stimuli; 187 target cues; 183 control non-target cues; 971 neutral stimuli.

### EEG RECORDING

The EEG was recorded continuously with NuAmp amplifiers (Neuroscan Inc., Albany, NY, USA) from 40 Ag/AgCl. Electrodes were positioned according to the 10–20 international system (AEEGS, 1991), including the following electrode positions: Fz, FCz, Cz, CPz, Pz, POz, Oz, FP1/2, F3/4, F7/8, FC3/4, FT7/8, C3/4, CP3/4, TP7/8, P3/4, P7/8, PO3/4, PO7/8, O1/2. Blinks and eye movements were monitored by deriving bipolar recording from an electrode placed below the right eye and FP2 (VEOG) and from electrodes placed to the left and right of the right eye (HEOG). The electrode in position AFz was used as the ground. The right mastoid was used as the active reference, but data were re-referenced off-line to the average of the left and right mastoids. Electrical impedance was kept below 5 kΩ and activity was filtered online with a low-pass filter of 300 Hz. The analog-to-digital sampling rate of the brain activity was set at 1000 Hz and data were recorded continuously for the entire experiment.

### PRE-PROCESSING OF EEG DATA

Data pre-processing was performed using EEGLab (Delorme and Makeig, 2004). The data were downsampled to 250 Hz, and then filtered with a high-pass filter at 0.1 Hz and low-pass at 45 Hz. Epochs started 200 ms before each stimulus onset and ended 1000 ms afterward. Average activity over 200 ms preceding the stimulus onset was used as a baseline against which all amplitudes were calculated. Noise, drift, artifacts, and blinks were excluded by manual inspection – corrupted epochs were excluded from further analyses. Data were then converted to Fieldtrip (Oostenveld et al., 2011) format for subsequent analyses.

### EEG ANALYSES

Event-related potentials (ERPs) were estimated for epochs extracted for the main experimental trial types: cued target; uncued target; cued control non-target; uncued control non-target; target cue; control cue; neutral. There were insufficient trial numbers to analyse invalid cueing conditions (e.g., neutral stimuli following predictive cues). Conditions of interest were averaged across eight blocks (each consisting of three consecutive runs, ∼380 trials) for regression analyses. For split-half analyses, conditions were averaged across two blocks (first half: runs 1 to 12 s half: runs 13–24). For the main analyses, we performed regression analyses to assess directly how the effect of probabilistic cueing varies over time. These were performed using a standard general linear model approach in MATLAB (see also Myers et al., 2014).

First, the EEG signal was averaged across pre-defined frontal (‘F3’,Fz’,‘F4’,‘FC3’,‘FCz’,‘FC4’), central (‘C3’,‘Cz’,‘C4’,‘CP3’, ‘CPz’,‘CP4’), and posterior (‘O1’,‘Oz’,‘O2’,‘PO3’,‘PO4’,‘POz’) clusters of electrodes (as in Figure 6 of Myers et al., 2014) in each of the eight blocks. We regressed the cue effect (cued target – uncued target; cued non-target – uncued non-target) estimated within each participant at each time-point from the eight consecutive (non-overlapping) blocks against block order (i.e., 1–8). This provides a parameter estimate (beta, in arbitrary units) that reflects the slope of the cue effect over time (i.e., a “learning effect”). The resulting betas constitute a learning-effect time-series for target learning and non-target learning. These beta parameters can be tested against zero for a basic effect of learning, and/or compared between conditions (see non-parametric tests below). Next, we also performed the equivalent learning-effect analysis on the cue-related response (target cue vs. neutral; non-target cue vs. neutral), and for completeness, both regression analyses were accompanied by a more straightforward half-way split of the data (first half vs. second half of the experiment). This provides a more intuitive, albeit coarser estimate of cue learning.

All group statistics were then performed using standard cluster-based non-parametric tests (Maris and Oostenveld, 2007). First, we estimate the *t*-statistic across participants for a contrast of interest at each time point, then define observed clusters of consecutive above-threshold time points, and calculated the cluster mass (by summing all *t*-values in an above-threshold cluster). Next, we randomly shuﬄe condition labels within participant (sign-flip for contrasts against zero), and extract the largest cluster mass produced by chance. This permutation step is performed 10,000 times to estimate the null distribution. The probability of the observed group-level cluster against chance is then derived as the rank order of the observed cluster relative to the null distribution (see also Nichols and Holmes, 2002; Myers et al., 2014). Significant effects were followed up with participant–wise correlations to behavior using Pearson correlation.

## RESULTS

### PREDICTED STIMULI: RESPONSE TO TARGETS AND CONTROL NON-TARGETS

The behavioral data revealed a benefit of target cueing (see **Figure 1C**). Reaction times were faster to cued targets relative to uncued targets (main effect cue: *F*_1,17_ = 4.701, *p* = 0.045, main effect block: *F*_7,119_ = 3.303, *p* = 0.003, interaction: *F*_7,119_ = 2.621, *p* = 0.015, cued minus uncued difference in first half of experiment: *t*_17_ = -1.320, *p* = 0.204, difference in second half: *t*_17_ = -2.211, *p* = 0.041), however, there was no effect of accuracy (main effect cue: *F*_1,17_ = 0.685, *p* = 0.419, main effect block: *F*_7,119_ = 0.994, *p* = 0.439, interaction:* F_7_*_,119_ = 1.357, *p* = 0.230). This behavioral evidence clearly suggests that participants could use the target-relevant cues to improve response times to the target stimulus, even though participants were not explicitly informed of the underlying predictive relationship. This benefit could arise because the inherent statistics of the sequence facilitate processing of any predictable stimulus, or because task-relevant predictions in particular are extracted and used to optimize behavior. By definition, there is no behavioral response to the non-target stimulus; therefore we must look to the neural data to adjudicate between these alternatives.

**FIGURE 2:**
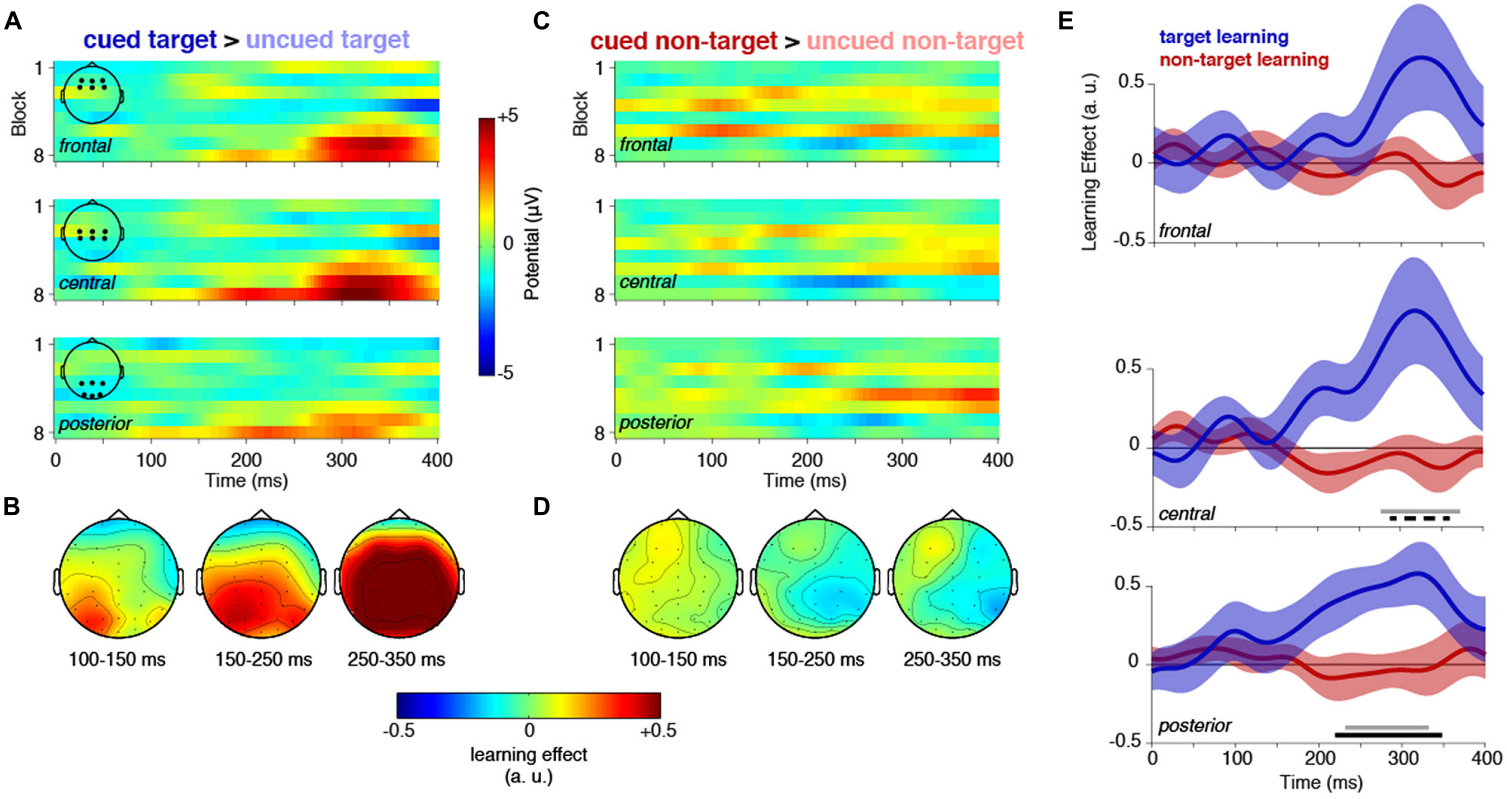
**Event-related potentials to cued vs. uncued targets and cued vs. uncued control non-targets. (A)** Target-related ERPs were modulated by target cues during learning. Plots show the mean potential difference between cued and uncued target stimuli, separately for each block, for three ROIs: frontal sensors (top panel, see inset for sensor locations), central (middle panel), and posterior (bottom panel). **(B)** Topography of learning effect. The topography shows the mean slope derived from the linear regression of task block onto potential difference, from three separate time-windows post target onset. **(C)** and **(D)** show the same as **(A)** and **(B)**, but for the control non-target stimulus. **(E)** The mean regression slope across the eight task blocks (fit separately at each time point) is shown for the same frontal, central, and posterior ROIs shown in **(A)** and **(C)**, for targets (blue lines) and non-targets (red lines). Shading indicates the standard error of the means (SEM). Horizontal bars indicate significant regression slopes in the target learning condition compared to chance (in black; central: *p* = 0.053, cluster-corrected, dashed line, posterior: *p* = 0.0130, cluster-corrected, solid line), and directly compared to the control non-target condition (in gray; central: *p* = 0.026; posterior: *p* = 0.045, cluster corrected).

First, we examined the EEG activity triggered by cued target stimuli relative to uncued targets (**Figure 2**). For illustration, difference voltages are plotted over the course of the experimental session in **Figures 2A,C**, but the statistical inference is drawn from the regression analysis in **Figure 2E**. Visual inspection of the difference plot (**Figures 2A,B**) reveals a cue-related positivity that was most evident in the central cluster of electrodes, emerging increasingly early in the trial toward the end of the experimental session. In contrast to the effects of learning task-relevant predictions, there was no evidence for a similar effect for the control non-target stimuli (**Figures 2C,D**).

Regression analyses confirmed a significant effect of target cueing (in blue) over time in central (time range: 288–360 ms, *p* = 0.052, cluster-corrected) and posterior sensors (time range: 220–348 ms, *p* = 0.013), but no corresponding effect of cueing the control non-target (in red). Importantly, the slope of the target learning effect was significantly larger than that for non-targets in the same time window, both at central (276–372 ms, cluster *p* = 0.026) and posterior sensors (232–332 ms, cluster *p* = 0.045).

This pattern closely resembles the behavioral performance over time (**Figure 1C**). In fact, *post hoc* analyses revealed a significant correlation between the neural effects (averaged between 250 and 350 ms) and the slope of the reaction time difference (between cued and uncued targets) across blocks (central sensors: *r*_17_ = -0.823, *p* = 2.7^∗^10^-5^, posterior sensors: *r*_17_ = -0.461, *p* = 0.054). However, in a speeded task, it is inherently difficult to tease apart activity associated with perception, decision-making and response preparation. Therefore, it is not possible in this experiment to pin-point the relative contribution of the cueing effect. It would seem likely that late effects in central/frontal electrodes reflect reduced latency of decision-making/response preparation (as evident in the behavioral profile **Figure 1C**), whereas the earlier cueing effects in posterior sensors might reflect modulations of perceptual processing. Although significant effects in our pre-defined posterior electrodes were only observed after 200 ms, there was a trend for relevant cue-related learning in more lateral posterior electrodes from approximately 100 ms (**Figure 2B**).

**FIGURE 3:**
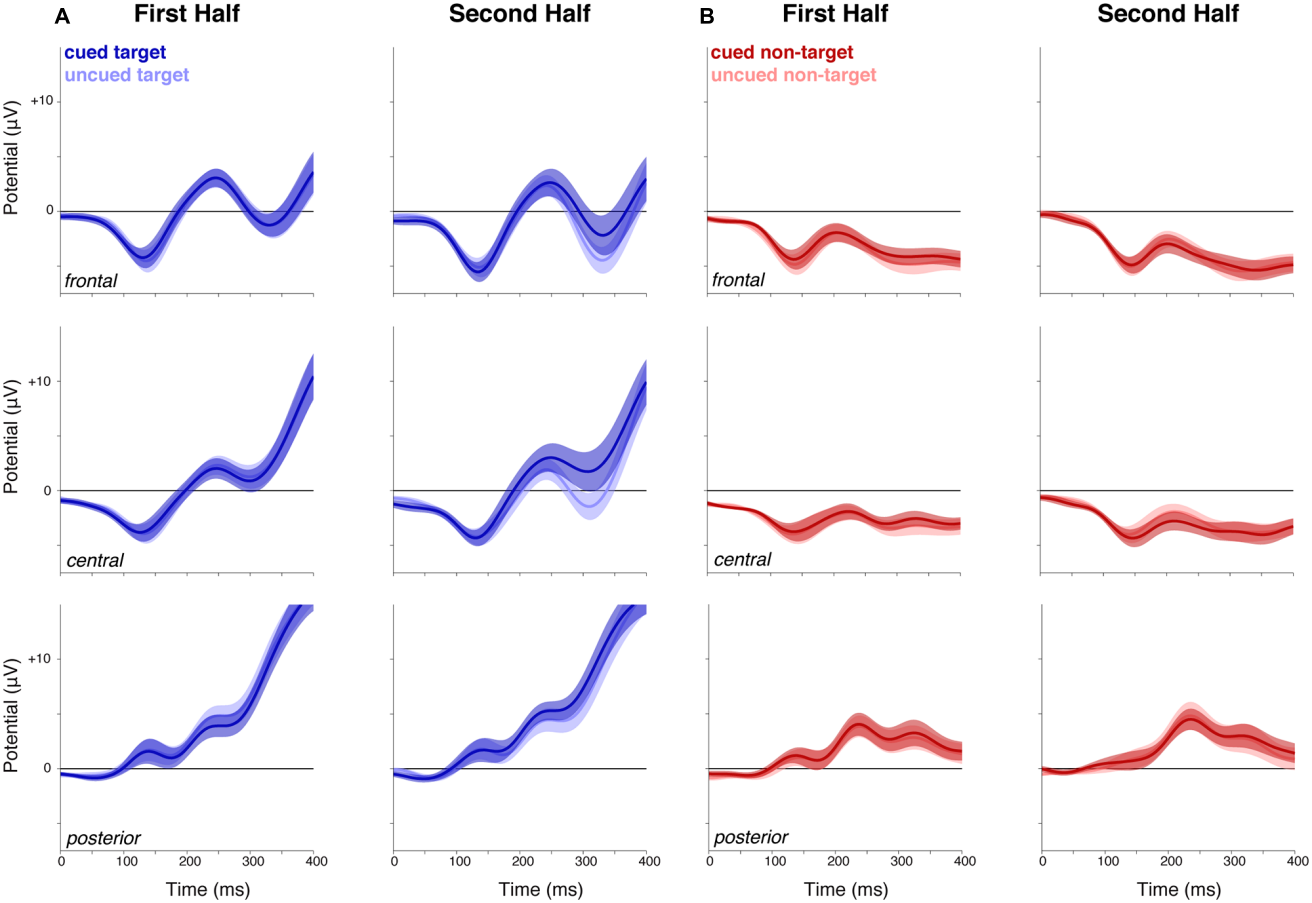
**Event-related potentials to cued and uncued targets and control non-targets, in the first and second half of the experiment. (A)** Cued and uncued targets. While there was no difference between cued and uncued target ERPs in the first half of the experiment (left panels), a positivity emerged between cued and uncued targets in the second half (right panels) at central and posterior sites. However, this did not survive cluster correction. **(B)** Cued and uncued non-targets. There was no evident difference between ERPs for cued and uncued non-targets. Shaded areas indicate SEM. ROIs are shown in **Figure 2**.

To visualize the results for central and posterior electrodes more clearly, the waveforms for cued/uncued target/control non-target stimuli are plotted for each half of the experimental session (**Figure 3**). These waveforms (**Figure 3A**) clearly show a cue-related positivity emerge in the second half of the experimental session at central and posterior electrodes, however, there were no significant clusters. For completeness, we also show the equivalent waveforms for the control non-target (**Figure 3B**).

### PREDICTIVE STIMULI: RESPONSE TO TARGET CUES AND CONTROL CUES

**FIGURE 4:**
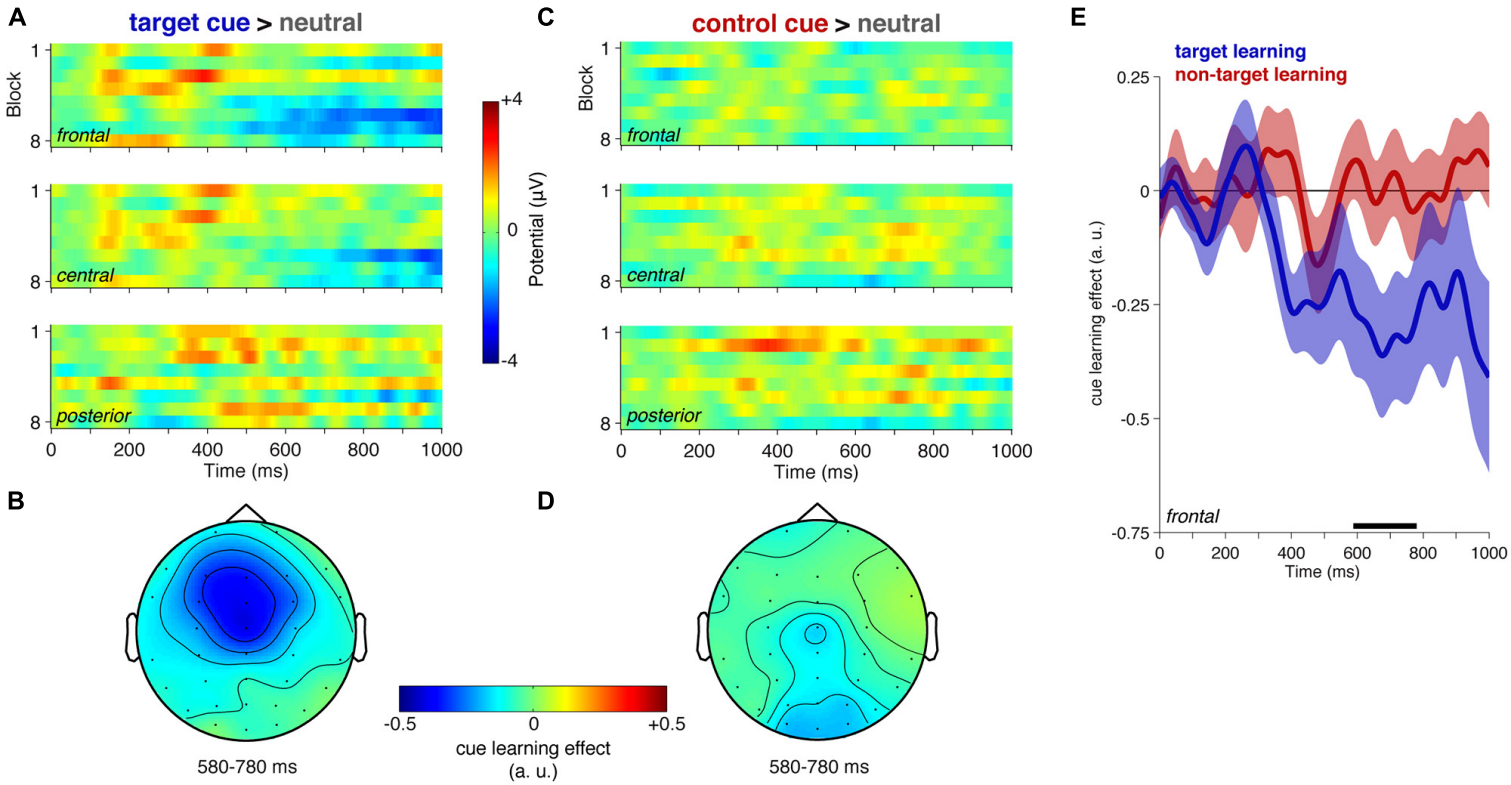
**Event-related potentials to predictive stimuli: target cue and control non-target cues. (A)** Plots show the mean potential difference between target cues and neutral stimuli, separately for each block, for the frontal, central, and posterior ROIs **(B)** Topography of learning effect. The topography shows the mean slope derived from the linear regression of task block onto potential difference. **(C)** and **(D)** show the same as **(A)** and **(B)**, but for the control cue. **(E)** The mean regression slope across the eight task blocks (fit separately at each time point) is shown for the frontal ROIs shown in **(A)** and **(C)**, for target cues (blue lines) and control cues (red lines). Shading indicates SEM. There was a significant effect of target cue learning (588–780 ms, *p* = 0.0343, cluster-corrected).

Next, we compared ERPs to the predictive stimuli: target-related cues and cues for control non-target stimuli. Again, plotting the ERPs over blocks throughout the experimental session, there is evidence for the development of a sustained negativity in frontal sensors by the end of the session (**Figures 4A,B**). This effect was evident as a statistically significant cluster in the regression of cue effect against block number that emerges just before 600 ms in frontal sensors (**Figure 4E**, 588–780 ms, *p* = 0.0514). Note, the negative relationship here is consistent with a negative cueing effect (cue < neutral) that increases in magnitude (i.e. gets more negative) over the course of the session (as illustrated in **Figure 4A**). The regression analysis did not reveal any other significant effects of the task-relevant target cue in the other electrode clusters. No significant effects were observed for the control cue (**Figures 4C,D**). The difference between the regression slope for target cues and control cues showed a trend, but was not significant (mean over 588–780 ms, *t*_17_ = -1.84, *p* = 0.083). *Post hoc* follow-up analysis of this frontal cueing effect revealed a significant correlation to behavior (*r*(17) = 0.582, *p* = 0.011).

**FIGURE 5:**
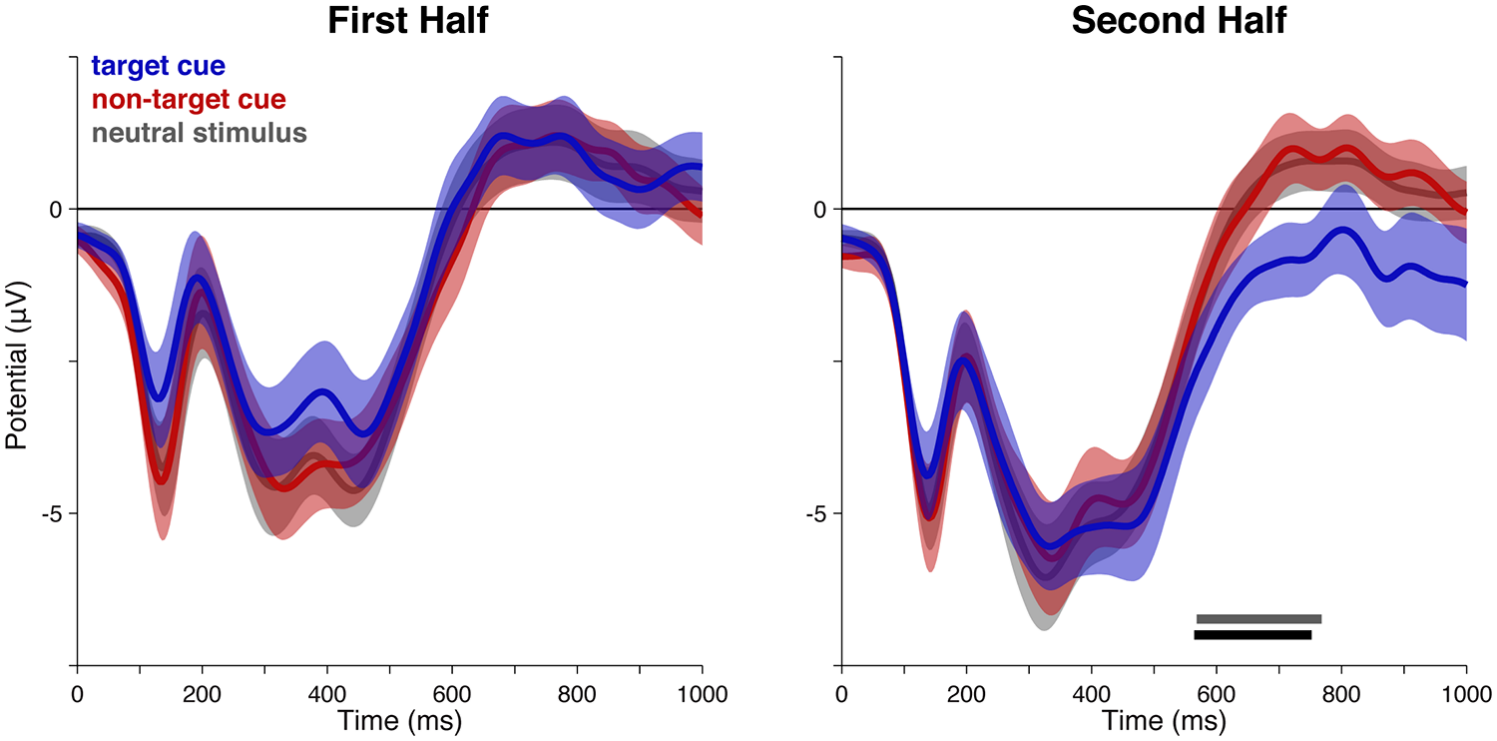
**Event-related potentials to target cues and control cues at frontal sensors in the first and second half of the experiment.** Conventions are the same as **Figure 3**. Horizontal bars indicate significant differences between the target cue relative to neutral (in black, *p* = 0.0039) and directly compared to the control non-target condition (in gray; one-tailed comparison cluster *p* = 0.0472).

The effect of the cue stimulus in frontal electrodes is clearly illustrated in **Figure 5**. In the second half of the experimental session, there is a robust negative potential that is specific for the task relevant cue, and is sustained until the onset of the next time (i.e., the likely target stimulus; 546–752 ms, cluster *p* = 0.0039). There is also a significant difference between target cue and control cue potentials in the second half of the session (568–772 ms, one-tailed comparison cluster *p* = 0.047), confirming that the learning effect was larger for target cues than for control cues (even though the regression effect above showed only a trend toward significance).

Interestingly, there was also a trend for an earlier positivity during the first half of the session (left panel in **Figure 5**; also see **Figure 4A** upper quadrant of the frontal plot). Although this did not reach statistical significance (subthreshold cluster in frontal learning effect at 380–452 ms, *p* = 0.1311), it is tempting to speculate that this early effect could reflect differential processing of the cue as participants start to learn that it might be task relevant. At this early stage, the predictive information is not yet used to guide behavior, but could reflect the initial stages of learning prior to later-stage exploitation of the statistical contingencies in the form of a sustained preparatory state (i.e., frontal negativity).

## DISCUSSION

The results of this experiment demonstrate that task-relevance critically influences how predictive relationships between sequential stimuli are learned and exploited for optimized behavior. Firstly, our behavioral results confirm that participants can learn predictable sequential relationships embedded in a stream of stimuli, and use this information to reduce reaction times to a designated target stimulus. This behavioral advantage was mirrored by an increased positivity in ERPs at central and posterior electrodes that emerged with learning, presumably reflecting enhanced processing for predicted targets. Importantly, EEG recordings also allowed us to track the neural response to the control non-target stimulus that was presented with identical statistical contingencies as the target. Despite the same predictive structure, we observed no neural effects of prediction for the non-target stimulus. Finally, we also identified a frontal negativity that was triggered by the learned predictive target cue, and that was sustained until the likely presentation of the target stimulus. Again, there was no equivalent neural effect for the control cue stimulus.

Prediction is often confused with task-relevance (Summerfield and Egner, 2009). In classic studies of attention, for example, cues typically provide predictive information related to a task-relevant target stimulus (i.e., target location). It is well established that under such circumstances, the response to validly predicted targets will be enhanced relative to invalidly cued (or unpredicted) targets (Posner, 1980). Early visual potentials are also amplified (Mangun et al., 1987). In contrast, a predictive coding framework maintains that the response to predicted stimuli should be reduced relative to unexpected input, as change provides more information about the environment than constancy (Rao and Ballard, 1999). These two features have often been cited as a fundamental conflict between core organizing principles in perception: attention and expectation (Spratling, 2008; Summerfield and Egner, 2009; Brown and Friston, 2013).

However, the apparent contradiction critically turns on the notion of task-relevance. Information implies a value-neutral quantity, but in ecological terms the utility of predictive information must co-depend on the value to behavior. The statistics of the environment allow the organism to predict events with potential consequences to behavior. Therefore, the expected utility of an event is determined by the inherent value of an event weighted by its probability. In a Posner cueing task, for example, the high-value stimulus (target) is weighted by the presentation probability: a cued location is more task-relevant to behavior because it has higher expected utility.

Here, we have shown that predictions for goal-related input trigger a distinct set of processes compared to predictions for irrelevant input. Reaction-time data confirmed the behavioral benefit of target cueing, even when participants were not explicitly instructed to adopt this strategy. The EEG data further confirmed an effect of target cueing. The response to cued targets was enhanced relative to uncued targets, presumably reflecting enhanced analysis of the target stimulus, decision-making and/or response preparation. Target-locked cueing effects were preceded by a sustained cue-related frontal negativity that presumably reflects preparation for the predicted task-relevant event. In contrast, we did not identify any significant effect of cueing the control non-target stimulus. These data clearly demonstrate that predictions for task-relevant event contingencies have a greater consequence than task-irrelevant statistical regularities. This is consistent with previous evidence that the statistical structure in the environment can be used to guide the focus of attention to likely task-relevant features (Chun, 2000; Summerfield et al., 2006).

Moreover, the current results are consistent with recent evidence that complex predictive relationships can also be learned implicitly (Zhao et al., 2013). In Zhao et al. (2013), sequences of task-irrelevant stimuli were presented at different spatial locations within in a standard visual search task. Unbeknownst to the participants, one of these locations contained a repeating triplet of stimuli. Although the structured sequence was completely task-irrelevant, spatial attention was drawn to the location of the predictable sequences, relative to random sequences. At first glance, this effect seems at odds with our lack of effect for task-irrelevant predictions. However, it should be noted that the predictive feature in Zhao et al. (2013) was shape, but the attention modulation was measured for location. In our study, attention was always in the same place, whereas we measured the neural response to relevant or irrelevant predicted stimuli. Perhaps more importantly, by including a task-relevant predictive relationship, we are able to show how predictions are influenced by task-relevance.

Previous studies of predictive coding often contrast predicted events against violations of expectation (e.g., den Ouden et al., 2009). Under these circumstances, it is difficult to disentangle the relative contribution of expectation suppression from an enhanced response to violations of expectation. In this study, we contrasted the effect of expectation against non-predictive stimuli in order to isolate more directly the contribution of expectation.

Although we found no robust evidence for neural modulation of task-irrelevant predictive cues, or the corresponding predicted non-targets, we cannot conclude that task-irrelevant predictions have absolutely no influence on brain and behavior. The key finding is a significant effect of task-relevance. It is always possible that we might have observed an effect for the task-irrelevant contingencies emerge if our methods were more sensitive or participants performed the task for longer. In the current task, learning was tracked over approximately one hour, but it is always possible that learning the irrelevant relationship in this task might have required further exposure. It is also worth considering the possibility that the irrelevant relationship was in fact learned relatively quickly, but was also quickly discarded because it could not be used to optimize behavior. In this scenario, both the task-relevant and irrelevant relationships might have been detected implicitly, but only the task-relevant contingency was maintained and used to optimize behavior. To test this hypothesis, it would be necessary to increase participant–wise statistical power to better track initial learning effects.

It is also worth noting that the predictive relationship employed in this study was relatively subtle. Firstly, most of the stimuli were not predictive (6 out of 10 neutral stimuli). Moreover, there were only two stimuli in a predictive sequence (compared to 3 in Zhao et al., 2013), and the predictive relationship was only 70% valid (compared to 100% in Zhao et al., 2013 and 80% valid in den Ouden et al., 2009). Evidently, this contingency was sufficient for subjects to learn and utilize if task-relevant, however, a stronger predictive relationship might have helped participants derive the irrelevant relationship. Again, this can only be addressed in future research.

Interestingly, a previous study by den Ouden et al. (2009) also found strong modulatory effects of task-irrelevant predictions using fMRI. In this case, task irrelevant audiovisual stimuli were paired with 80% validity. Although participants reported no awareness of this predictive relationship, the response to the predicted visual stimulus was modulated as a function of learning. It is difficult to relate these results directly to our experiment because they included no task-relevant predictive relationship for comparison. However, one could speculate that the effect of expectation suppression was particularly robust in their study because the predictive stimuli were more distinctly task-irrelevant. Task-irrelevant predictions were manifest in stimuli presented prior to the task-relevant phase of the trial (i.e., target presentation). Therefore, participants could easily demarcate these stimuli as task-irrelevant, which might be important for suppressive effects of expectation.

In contrast, predictive structure in the current experiment was embedded within a stream of stimuli that were all potentially task-relevant. Therefore, suppressive effects might have been masked by the overall attentional focus on the task-relevant stream of stimuli. To address this question properly, it would be necessary to disentangle the task relevance of the prediction from the task relevance of the stimuli and behavioral context. Finally, it is also worth noting that the stimuli used in our study were all suprathreshold, therefore we focused on reaction-time effects rather than changes in perceptual sensitivity. Our choice of stimuli could also have had implications for our EEG results, reducing the opportunity to observe early perceptual modulations, including suppression or enhancements. Indeed, it is likely that the neural consequences of prediction depend on the type of information they reveal within the context of the task and the processing limits.

In summary, the results reported here show that task relevance modulates how implicit regularities in the environment are extracted, learned and exploited for goal-relevant behavior. We suggest that the utility of statistical predictions critically depends on the relationship to behaviorally relevant events, and how they can be used to optimize behavior within the context of task demands.

## Conflict of Interest Statement

The authors declare that the research was conducted in the absence of any commercial or financial relationships that could be construed as a potential conflict of interest.
